# Functional Connectivity Patterns and the Role of 5-HTTLPR Polymorphism on Network Architecture in Female Patients With Anorexia Nervosa

**DOI:** 10.3389/fnins.2019.01056

**Published:** 2019-10-14

**Authors:** Enrico Collantoni, Paolo Meneguzzo, Marco Solmi, Elena Tenconi, Renzo Manara, Angela Favaro

**Affiliations:** ^1^Department of Neurosciences, University of Padua, Padua, Italy; ^2^Padova Neuroscience Center, University of Padua, Padua, Italy; ^3^Radiology Unit, Department of Medicine and Surgery, Neuroscience Section, University of Salerno, Salerno, Italy

**Keywords:** eating disorders, anorexia nervosa, neuroimaging, graph theory, fMRI, resting state, 5-HTTLPR

## Abstract

**Introduction:**

Recent neuroimaging studies suggest that anorexia nervosa (AN) symptoms emerge from failures in the relationships between spatially distributed networks that support different cognitive, emotional, and somatosensory functions. The 5-HTTLPR genotype has been shown to modulate all these abilities in AN, as well as the connectivity patterns between brain regions that support their functioning. This study aims at exploring the presence of any difference in functional connectome properties between AN patients and healthy controls (HC) by means of graph theory tools. The effect of 5-HTTLPR genotype on regional and global network characteristics in AN and HC was also explored.

**Methods:**

A sample of 74 subjects (38 HC, 36 AN) underwent a resting state functional magnetic resonance imaging and was genotyped for 5-HTTLPR polymorphism. Comparisons of network properties were made between the AN and HC groups and, within each group, between 5-HTTLPR carriers of low-functioning alleles and carriers of the long–long genotype.

**Results:**

Patients with AN displayed lower network clustering than HC (*p* = 0.04 at Mann–Whitney *U* test). Based on both degree and betweenness, a different distribution of network hubs emerged in the two groups. In particular, the anterior part of the anterior cingulate cortex was a hub only in the patient group. A correlation emerged between differences in brain volumes between patients and HC and differences in degree values of basal ganglia, nodes in the insula, and those in the parietal cortex. Carriers of the short allele of the 5-HTTLPR polymorphism were characterized by lower small-world properties (*p* = 0.027) and modularity (*p* = 0.031) in the patient group, and a trend toward higher modularity (*p* = 0.033) and small-world values (*p* = 0.123) in the HC group.

**Discussion:**

Patients with AN showed differences in hubs distribution, providing evidence of the presence of a different functional architectural backbone in this group. Since some correlation emerged between different degree values of nodes and differences in volumes, further longitudinal studies are warranted to better understand the role of malnutrition on brain network architecture. The opposite effects of 5-HTTLPR polymorphism on global network characteristics in the two groups suggest an interaction of the short allele and malnutrition in modulating brain network properties.

## Introduction

In recent years, studies that evaluate the neurobiological underpinnings of anorexia nervosa (AN) have greatly increased and revealed that this disorder is characterized by complex and multifaceted patterns of interaction between genetic, neuropsychological, and connective characteristics (Zipfel et al., 2015; Solmi et al., 2018; Frank et al., 2019).

Within neuroimaging research, most of the studies in AN are aimed to explore the neural processing of stimuli that activate specific processes (i.e., taste or reward processes), as well as the functional relationships between different areas during rest (Fuglset et al., 2016; Gaudio et al., 2016). Results of these studies are extremely heterogeneous, due to the complexity of the disorder and often different study protocols and designs. Nevertheless, a common point of all these observations is that brain functioning in AN is not characterized by isolated dysfunction in circumscribed brain areas but rather from disturbance in spatially distributed neural networks that support several functions and processes (Steinglass et al., 2018; Seidel et al., 2019).

The possibility to describe the interactions between anatomically distributed areas have been greatly supported, in recent years, by the application of graph theory tools to neuroimaging data (Bullmore and Sporns, 2009). Graph theory allows the mathematical representation of the brain as an architecture of nodes, which are represented by discrete brain areas, and edges, which represent the functional or structural correlations between the nodes. The advantage of this framework is its ability to describe the organizational principles that govern the interactions between different brain regions and to analyze the relevance that specific areas have in managing the connectivity within a network (Rubinov and Sporns, 2010).

To date, three studies evaluated brain connectivity in AN by means of graph theory tools but have considered very different samples. The first compared a sample of acutely ill patients with healthy controls (HC), and pointed out both global and regional anomalies. Regionally, patients with AN showed reduced connectivity strength in insula and a lower centrality of thalamus. Globally, they showed higher levels of separation between nodes in the overall network as well as a higher tendency of nodes with similar connectedness to link together (Geisler et al., 2016). The second one compared a sample of patients recovered from AN and HC and pointed out the presence, in the experimental group, of a higher tendency of the network to form clusters of densely interconnected nodes (increased clustering coefficient) and a higher tendency of nodes with similar connectedness to link together (increased assortativity). Furthermore, patients with AN reported a different balance between segregation and integration properties when compared to HC [reduced small-world index (SWI)] (Geisler et al., 2018). The third study was conducted on a sample of adolescent patients at first stages of AN and pointed out the presence of a decreased connectivity in a sub-network of connections that encompass the left and right rostral anterior cingulate cortex, the left paracentral lobule, the left cerebellum, the left posterior insula, the left medial fronto-orbital gyrus, and the right superior occipital gyrus in the experimental sample (Gaudio et al., 2018).

Alongside the evaluation of the rules that govern the relationships between different brain areas in the connectome, graph theory allows the identification of those regions of the brain that have a peculiar role in supporting network communication and integration and that are called “hubs” (van den Heuvel and Sporns, 2013; Oldham and Fornito, 2019). The ability of hub regions to mediate large proportion of communications in the brain, as well as their tendency to integrate distributed neural signals, makes them susceptible to different disease processes. Coherently with these observations, network hubs have been demonstrated to be points of vulnerability into a network, and alterations in their distribution have been evidenced in many neurologic and psychiatric disorders like depression, ADHD, schizophrenia, and Alzheimer disease (Rubinov and Bullmore, 2013; Dai et al., 2014; Hong et al., 2014; Korgaonkar et al., 2014).

No study to date investigated the presence of any alteration in hubs distribution in patients with AN. The evaluation of hubs distribution in AN could be particularly interesting both from a neurobiological and from a clinical point of view. In fact, an alteration or a disproportion in their distribution could help in identifying specific areas of vulnerability in the disorder as well as in better characterizing the functional correlates of specific cognitive dysfunctions and psychopathological dimensions.

Another point of interest, in the evaluation of network properties in AN, seems to be the investigation of the role of genes in mediating the configuration of the connectome. In fact, the presence of specific polymorphisms, like the 5-HTTLPR polymorphism for the serotonin transporter gene, have been evidenced to modulate the connectivity patterns in different functional networks in AN (Collantoni et al., 2016). Furthermore, in AN, the 5-HT modulation have been proposed to be involved in contributing to specific psychopathological and temperamental dimensions, in regulating satiety and food consumption, and in determining cognitive and executive functioning (Haleem, 2012; Tenconi et al., 2016). Thus, the evaluation of the role of serotoninergic circuits in modulating the architectural properties of the functional brain connectome in AN can help in better characterizing their role in the neurobiology of the disorder as well as their possible relevance as targets of serotoninergic drugs.

Our purpose in this study is therefore to explore the presence of any difference in functional connectome properties between AN patients and HC by means of graph theory tools. The effect of 5-HTTLPR genotype on regional and global network characteristics in AN and HC was also examined. We hypothesized to find differences between patients and controls in assortativity values and hubs distribution. We also hypothesize the presence of a relationship between alterations in morphology of brain areas and their alterations in network properties, as observed in other psychiatric disorders (Crossley et al., 2014). Finally, we hypothesize to find effects of the 5-HTTLPR polymorphism that are similar in cases and controls.

## Materials and Methods

A total of 38 patients with acute AN and 38 HC were included in this study.

Patients with AN were recruited from the Padova Hospital Eating Disorders Unit. All the subjects of the experimental group met DSM-5 criteria for AN. A sample of HC was recruited from the same geographical area. The HC group was similar to the patient group in age, ethnicity, educational level, and hand lateralization. Exclusion criteria for recruitment in both experimental and HC groups were male gender, history of head trauma or injury with loss of consciousness, history of any serious neurological or medical illness, active use of systemic steroids, pregnancy, active suicidality or major depression, history of substance/alcohol abuse or dependence, bipolar disorder or schizophrenia spectrum disorder, moderate mental impairment (IQ < 60) or learning disabilities, use of medications other than antidepressants, and known contraindications to conventional MRI. In HC, history of any psychiatric disorder and any first-degree relatives with an eating disorder were considered additional exclusion criteria. After recruitment, we excluded 2 AN patients for technical reasons (see below).

Table 1 describes the main characteristics of the sample. Thirteen patients with AN were under drug treatment with antidepressants at the time of scanning (1 patient mirtazapine, 2 paroxetine, 2 escitalopram, 1 fluoxetine, and 7 sertraline).

**TABLE 1:** Baseline characteristics of the two groups.

	**Patients with AN (*n* = 36)**	**Healthy women (*n* = 38)**	**Mann–Whitney *U* test**
	**Mean (SD)**	**Mean (SD)**	***p***
Age (years)	26.0 (7.0)	25.3 (6.3)	0.729
Age at onset (years)	18.6 (5.0)	=	=
Duration of illness (months)	73.3 (79.2)	=	=
Baseline BMI (kg/m^2^)	15.8 (1.8)	21.7 (2.9)	0.000
Lowest BMI (kg/m^2^)	14.0 (1.8)	19.8 (2.5)	0.000
Edinburgh laterality index	58.2 (37.2)	55.0 (42.0)	0.541
WCST global index	48.0 (38.6)	42.2 (29.6)	0.995
WCST number persev. responses	16.9 (16.4)	12.4 (7.4)	0.979
Rey central coherence index	0.98 (0.43)	1.16 (0.39)	0.101
Rey visual memory	17.5 (5.4)	20.6 (4.6)	0.026
Iowa gambling task net score	3.6 (27.1)	21.5 (29.5)	0.009
	*N/tot* (%)		Pearson χ^2^
5-httlpr short allele	29/36 (81%)	26/38 (69%)	1.43 (0.23)
Restricting subtype	26/36 (72%)	=	=

Ethical permission was obtained from the Ethics Committee of the Hospital of Padova. After completely describing the study to the subjects, written informed consent was obtained.

### Clinical and Neuropsychological Assessment

All subjects were investigated for AN diagnosis with a diagnostic interview according to the Eating Disorders Section of the Structured Clinical Interview for Diagnostic and Statistical Manual of Mental Disorder (DSM-5, American Psychiatric Association, 2013) and, also, a semi-structured interview was used in order to collect socio-demographic and clinical variables (Favaro et al., 2012, 2013). More information about subjects’ psychopathology was achieved using the Hopkins Symptoms Checklist (Derogatis et al., 1974), the Eating Disorders Inventory (Garner et al., 1983), and the State–Trait Anxiety Inventory (Spielberger et al., 1999). Furthermore, the Edinburgh Handedness Inventory (Oldfield, 1971) was used to assess handedness (and left-handed individuals were excluded).

The neuropsychological assessment included the Wisconsin Card Sorting Task (WCST) and measures of intellectual abilities. WCST is one of the most widely used measures assessing abstract thinking and set-shifting abilities (Berg, 1948). The global score (a general measure of executive functioning) and number of perseverative responses (a measure of cognitive inflexibility) were used as main outcomes (Tenconi et al., 2010). Subjects were also assessed with the Rey–Osterrieth Complex Figure Test in order to assess participants’ memory, visual–spatial abilities, and central coherence. Outcomes from this test is the Central Coherence Index (CCI), calculated from the style index and the copy order index, and the Visual Memory Index, calculated from the correct reproduction of the 18 items of the original figure (Tenconi et al., 2010). To exclude mental impairment, all participants over the age of 20 completed the Brief Intelligence Test (TIB), which measures premorbid intellectual ability (the test is very similar to the National Adult Reading Test for the Italian population) (Colombo et al., 2002). Participants younger than 21 completed the Information subtest of Wechsler Intelligence Scale (the children version if participants aged 16 or less, and the adult version if their age was between 17 and 20 years) as a measure of verbal intelligence (Wechsler, 1949).

### Data Acquisition

Data were collected on a Philips Achieva 1.5-T scanner equipped for echo-planar imaging. A resting-state fMRI scan entailed 250 continuous functional volumes (repetition time = 2009 ms, echo time = 50 ms, flip angle = 90°, 21 slices, matrix = 128 × 128, acquisition voxel size = 1.8 × 1.8 × 6 mm, acquisition time = 8 min; field of view = 23 cm). Participants were instructed to rest with their eyes closed during the scan. High-resolution 3D T1-weighted anatomical images were also acquired in a gradient-echo sequence (repetition time = 20 s, echo time = 3.78 ms, flip angle = 20°, 160 slices, acquisition voxel size = 1 × 0.66 × 0.66 mm, field of view = 21–22 cm).

### Data Processing and Statistics

Structural images were preprocessed using the FreeSurfer package (Martinos Center for Biomedical Imaging, Massachusetts General Hospital, Boston) version 5.3.0. The cortex was then divided into 148 regions of interest (ROIs) (74 per hemisphere) with a specific sulco-gyral atlas (Destrieux atlas) (Destrieux et al., 2010). The 148 cortical regions and the subcortical nuclei volumes (caudate, putamen, accumbens, hippocampus, amygdala, palladum, and thalamus) obtained were then transformed and warped using a MNI_2 mm map, summed to build a probability map of every single brain areas, and then transformed in functional space using FLIRT. Seed ROIs were obtained by thresholding probability maps at 80%.

Resting-state scans were preprocessed with both Analysis of Functional NeuroImages (version AFNI_2010_10_19_1028^1^; NIMH, Bethesda, Maryland) and FM-RIB Software Library (version FSL 4.1.6^2^; FMRIB, Oxford, United Kingdom).

Signal-to-noise ratio was computed as described by van Dijk et al. (2012) for each resting-state sequence and used to estimate data quality. Only resting-state scans with a mean slice-based temporal signal-to-noise ratio higher than 100 were included in the subsequent analyses. All but two scans (two patients with AN) passed this criterion.

Preprocessing was then performed as described in our previous reports (Favaro et al., 2013, 2014a,b). Details of preprocessing and processing are described in the Supplementary Information. A high-pass filter setting of 200 s (0.005 Hz) was used to reduce very-low-frequency artifacts such as scanner drift and a low-pass filter to remove any components in the high-frequency spectrum (40.1 Hz).

A seed-based approach was used to explore the functional connectivity of brain areas obtained as described before. Nuisance signals were removed by multiple regression before functional connectivity analyses. Each individual’s 4D time series were regressed on nine predictors, consisting of white matter, cerebrospinal fluid, the global signal, and six motion parameters (three cardinal directions and rotational movement around three axes). The time series of the nuisance signals were extracted by (a) averaging all voxels in the brain (global signal) across the time series; (b) segmenting each individual’s high-resolution structural image (FAST, FSL) (Zhang et al., 2001), applying a threshold at 80% tissue type probability, and averaging all voxels within the thresholded mask (white matter and cerebrospinal fluid) across each time series; and (c) using the residuals obtained after motion correction by MCFLIRT (FMRIB, Oxford, United Kingdom). Each subject’s residual 4D time series was transformed into Montreal Neurological Institute space by means of a linear affine transformation implemented in FSL (FLIRT) and the time series extracted for each seed.

Analyses of network properties were performed with Graph Analysis Toolbox (GAT; Stanford University School of Medicine, Stanford, CA, United States) (Hosseini et al., 2012). Undirected weighted networks were constructed by averaging time series across all voxels in the seed ROI. A 148 × 148 association matrix was constructed for each individual by means of pairwise Pearson correlation coefficients. Each entry of the symmetric correlation matrices represents the strength of functional connectivity between two ROIs. By thresholding the correlation values between matrix entries, an adjacency matrix is derived for each association matrix. In particular, a range threshold of 0.1–0.5 with increments of 0.05 was applied in order to estimate the binary adjacency matrices. The statistical differences between groups in the network measures, which are quantified for each individual network, were determined by means of a non-parametric permutation test with 1000 repetitions. In addition to comparing global network measures at every density, AUC analyses were performed to make the between-group comparison less sensitive to the thresholding process.

### Graph-Based Metrics

Segregation and integration are two major organizational principles of brain structure and function. Segregation properties of a network describe its tendency to be composed by specialized and functionally coherent areas. Parameters that describe the segregation of a network are the clustering coefficient (that indicate the density of connections between the neighbors of an individual node) and the modularity (that measures the correlation between the probability of having an edge that connect two nodes and the probability that nodes are part of the same community).

Integration properties of a network describe its ability to manage a globally distributed and efficient communication. Parameters that describe the integration of a graph are the global efficiency (which measures the efficiency of information transfer across the network, where maximal GE values indicate a fully connected network) and the characteristic path length (which indicates the number of edges that are present in the shortest path of two nodes, averaged over all pairs of nodes).

Assortativity is the correlation between the degrees of connected nodes and reflects the tendency of a brain region to connect with nodes of similar degree (Boccaletti et al., 2006; Rubinov and Sporns, 2010).

A network is small world if high clustering coexists with high efficiency. This means that small-world networks combine the ability to use a relatively small number of long-distance connections to synchronize the information flow and the advantage to use local connections to locally processing information. Therefore, the SWI is computed by comparing the CPL and the CF of a graph with the corresponding values of null random graphs with same number of nodes, edges, and degree distribution (Bassett and Bullmore, 2006).

Hubs are crucial regulators of information flow across the network and play a key role in network resilience to insult. Degree and betweenness centrality identify hub nodes by measuring the fraction of short paths between nodes of the network that pass through a given node. Nodes that are characterized by high degree and/or betweenness centrality values are likely to participate in many of the network’s short paths and to have a great control over the flow of information within the network (van den Heuvel and Sporns, 2013).

### Genetic Analysis

DNA samples were collected at the time of assessment in all participants. Participants were genotyped for the presence of the short variant of the 5-HTTLPR gene, and the A/G single-nucleotide polymorphism (SNP rs25531) of the 5-HTTLPR gene, according to previously described standard protocols (Favaro et al., 2014a). For the 5-HTTLPR gene, samples were split on the basis of the presence or absence of the short variant (S) of the 5-HT transporter, and polymorphism G were included in this short group variant.

### Statistical Analyses

Global and local graph metrics is usually considered not normally distributed (Hosseini et al., 2012). For this reason, for between-group comparisons of graph metrics, non-parametric independent two-group Mann–Whitney *U* tests with a critical *p* value of 0.05 were performed. Given the explorative nature of the study, we decided to avoid correction for multiple comparisons for global graph metrics. However, to reduce the possibility of false-positive results, we chose to consider only six parameters of the available graph metrics. Local graph metrics were, on the contrary, corrected for multiple comparisons regarding the number of nodes with FDR.

## Results

### Comparison Between Patients and Healthy Women

**FIGURE 1:**
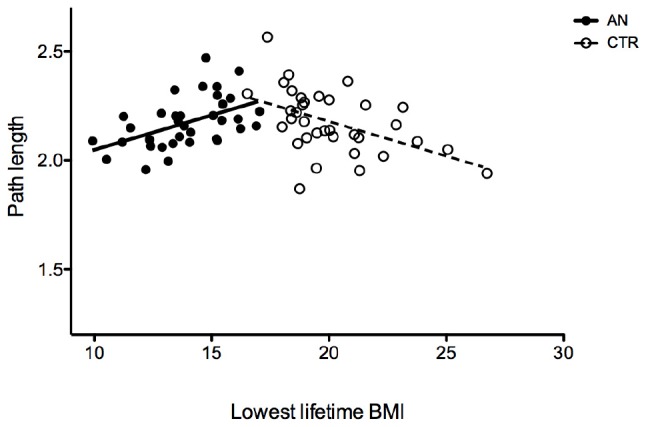
Correlation between the lowest BMI lifetime and characteristic path length in patients with AN and HC.

Patients with AN showed no differences when compared to HC in whole-brain segregation and integration measures, except for a slightly lower clustering score (*p* = 0.04 at Mann–Whitney *U* test; Table 2). Measures of net integration/segregation did not show any significant correlation with age, body mass index (BMI), age of onset, duration of illness, eating psychopathology, and SCL depression or obsessive–compulsive scores. The lifetime lowest BMI showed a significant positive correlation with path length (rho = 0.42; *p* = 0.011) in the group of AN patients and a negative one (rho = –0.42; *p* = 0.011) in the control group (Figure 1). In patients with AN, the CCI (Rey Figure Task) correlated with clustering values (rho = 0.42; *p* = 0.012), whereas both state and trait STAI scores negatively correlated with global efficiency (rho = –0.56; *p* = 0.001; rho = –0.39; *p* = 0.019). No differences in net measures emerged comparing diagnostic subtype groups, or comparing patients who were on antidepressant treatment and those who were not.

**TABLE 2:** Network properties and hubs distribution in AN patients and healthy women.

	**Patients with AN (*n* = 36)**	**Healthy women (*n* = 38)**	**Mann–Whitney *U* test**
	**Mean (SD)**	**Mean (SD)**	***p***
Assortativity	0.29 (0.11)	0.33 (0.14)	0.187
Global efficiency	0.49 (0.06)	0.48 (0.05)	0.443
Clustering	0.61 (0.04)	0.62 (0.04)	0.034
Modularity	0.28 (0.09)	0.31 (0.09)	0.217
Path length	2.16 (0.16)	2.16 (0.16)	0.736
Sigma	1.34 (0.32)	1.41 (0.26)	0.284
Net hubs betweenness	Anterior part of the left cingulate gyrus and sulcus	Left superior frontal gyrus	
	Left precuneus	Left precuneus	
	Lateral aspect of the left superior temporal gyrus	Lateral aspect of the left superior temporal gyrus	
	Right superior frontal gyrus	Right superior frontal gyrus	
	Right precuneus	Right precuneus	
	Lateral aspect of the right superior temporal gyrus	Lateral aspect of the right superior temporal gyrus	
		Right middle temporal gyrus	
Net hubs degree	Anterior part of the left cingulate gyrus and sulcus	Left transverse frontopolar gyri and sulci	
	Left middle frontal gyrus	Left parahippocampal gyrus	
	Left subcallosal area	Left subcallosal area	
	Right middle frontal gyrus	Right parahippocampal gyrus	
	Right subcallosal area	Right subcallosal area	
		Posterior segment right lateral sulcus	

**FIGURE 2:**
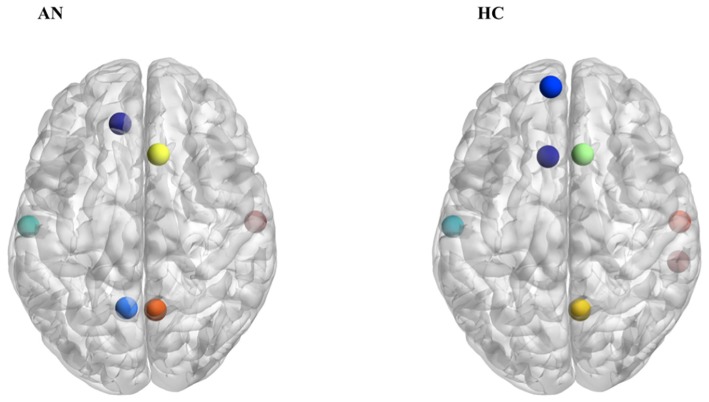
Hubs distribution based on betweenness values in patients with AN and HC.

**FIGURE 3:**
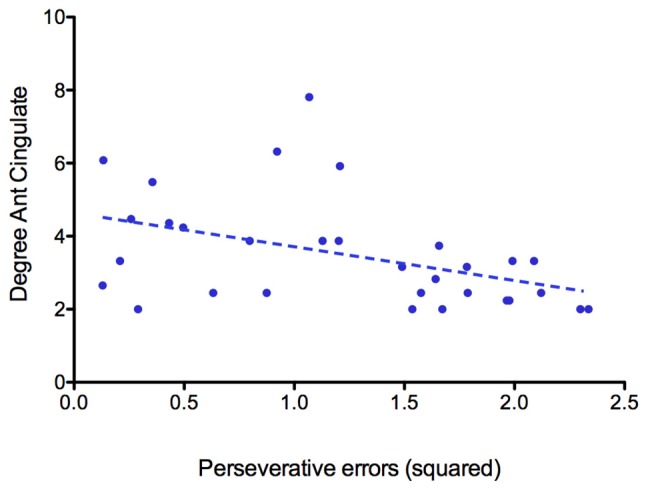
Correlation between the degree of the anterior part of the anterior cingulate gyrus and the number of perseverative errors at WCST.

Based on both degree and betweenness values, a partially different distribution of network hubs emerged in patients with AN and HC (Table 2). In particular, analyzing betweenness, the left superior frontal gyrus was lacking in the AN group, while the anterior part of the cingulate gyrus represented a hub region only in patients with AN (Figure 2). For degree values, AN showed a higher betweenness value for the anterior part of the cingulate gyrus and right/left middle frontal gyri, whereas in healthy women, hubs were located in bilateral parahippocampal gyri, in the left transverse frontopolar gyri and in the posterior segment of the right lateral sulcus. The degree of the anterior part of the cingulate gyrus significantly negatively correlates (*p* = 0.014; *R*^2^ = 0.19) with the number of perseveration errors at the WCST (Figure 3).

**FIGURE 4:**
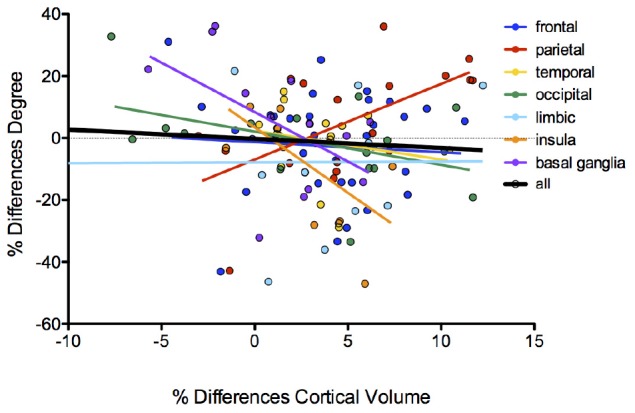
Correlation between brain volumes changes and degree/betweenness values of brain nodes.

Testing the hypothesis of a relationship between brain volume decrease and changes in degree/betweenness values of brain nodes (Figure 4), we found trends for decreased values of degree along with increased differences in volumes in the comparison between patients and controls in nodes of the insula cortex (*p* = 0.033; *R*^2^ = 0.38) and in basal ganglia (*p* = 0.05; *R*^2^ = 0.28). On the contrary, nodes of the parietal cortex showed an inverse relationship: increased degree values along with increased differences between patients and controls in both volume (*p* = 0.01; *R*^2^ = 0.35) and thickness (*p* = 0.018; *R*^2^ = 0.30; Supplementary Figure 1) of the cortical areas. No significant correlation emerged for betweenness values. No significant differences emerged in regional network measures between patients with AN and HC. No differences emerged on graph metrics between patients who were taking antidepressants and those who did not (Mann–Whitney *U* test: global efficiency: *p* = 0.43, clustering coefficient: *p* = 0.54, modularity: *p* = 0.54, characteristic path length: *p* = 0.17, SWI: *p* = 0.41, assortativity: *p* = 0.90).

### 5HTTLPR Polymorphism Effects on Network Measures

According to 5-HTTLPR polymorphism, in patients with AN, the low functioning genotype was associated with significantly lower small-world properties and lower modularity compared with the high functioning LL genotype (Table 3). On the contrary, in HC, the group who carried the S allele showed significantly higher modularity and a trend toward higher values of small-worldness in comparison with the LL genotype (Table 4).

**TABLE 3:** Network properties in patients with AN according to 5-HTTLPR genotype.

	**Patients with AN S allele (*n* = 29)**	**Patients with AN LL genotype (*n* = 7)**	**Mann–Whitney *U* test**	**Cliff’s delta**
	**Mean (SD)**	**Mean (SD)**	***p***	**Effect size**
Assortativity	0.30 (0.12)	0.30 (0.10)	0.845	0.054
Global efficiency	0.48 (0.05)	0.49 (0.08)	0.410	0.212
Clustering	0.61 (0.04)	0.62 (0.05)	0.424	0.079
Modularity	0.27 (0.09)	0.35 (0.06)	0.031	0.448
Path length	2.18 (0.12)	2.10 (0.27)	0.838	0.034
Sigma	1.29 (0.29)	1.64 (0.32)	0.027	0.517

**TABLE 4:** Network properties in HC according to 5-HTTLPR genotype.

	**Healthy women S allele (*n* = 26)**	**Healthy women LL genotype (*n* = 12)**	**Mann–Whitney *U* test**	**Cliff’s delta**
	**Mean (SD)**	**Mean (SD)**	***p***	**Effect size**
Assortativity	0.35 (0.15)	0.29 (0.12)	0.207	0.263
Global efficiency	0.48 (0.06)	0.49 (0.05)	0.958	0.006
Clustering	0.63 (0.03)	0.61 (0.03)	0.198	0.263
Modularity	0.33 (0.09)	0.28 (0.09)	0.033	0.359
Path length	2.15 (0.19)	2.18 (0.10)	0.930	0.103
Sigma	1.45 (0.25)	1.30 (0.27)	0.123	0.276

## Discussion

The present study evidences the presence of specific alterations in the configuration of functional connectivity architecture in AN. The experimental group showed, when compared to the HC group, a different distribution of hub regions based on both betweenness and degree values. The importance of evaluating hub regions in brain disorders is twofold, as these areas not only are crucial in supporting the network communication but also represent points of vulnerability to several pathogenic processes affecting the brain (Fornito et al., 2017). Therefore, the observation of a different distribution of hubs into the functional connectome in patients with AN does not have a univocal interpretation, being able to reflect their functional relevance in the overall connectome as well as the pathological consequence of disorder-related processes.

Our results pointed out that the hubs distribution between patients with AN and HC differs for some nodes, while others are identically distributed between the two groups. Interestingly, the main differences are identifiable mostly in a disproportion between frontal and subcortical areas, the former being more represented in AN patients, while the latter are more expressed in the control group. In particular, the left anterior cingulate gyrus is present only in patients with AN when hubs are computed on both betweenness and degree values. Hub regions are considered to offer an architectural backbone for brain processes that require high levels of functional integration, like cognitive and executive functions (Crossley et al., 2014). Therefore, the peculiar hubs distribution in patients with AN may represent a fundamental component of the functional connective architecture that supports the cognitive functioning in the disorder. The prevalence of frontal regions over subcortical ones in our experimental group may be coherent with this hypothesis, since it may sustain the presence of unbalanced top-down and bottom-up processes in AN (O’Hara et al., 2015). In AN, the anterior cingulate gyrus has been shown to have an important role in performance monitoring and in the cognitive regulation of appetitive stimuli, and it has also been proposed to be functionally involved in set shifting abilities as well as in cognitive inflexibility (Kaye et al., 2013; Geisler et al., 2017). The observation that this node is highly connected in patients with AN but not in HC confirms the hypothesis, already supported by previous observations, that it plays a crucial role in AN neurobiology and psychopathology (Kuyck et al., 2009). The negative correlation between the degree of anterior cingulate gyrus and perseverative errors at WCST suggests that this area may be involved in the ability to modulate cognitive and behavioral flexibility in AN. Overall, these observations suggest a possible role of cognitive training protocols (i.e., cognitive remediation therapy) in the treatment of AN. Furthermore, brain-directed treatments (i.e., repetitive transcranial magnetic stimulation or transcranial electrical stimulation) that can modulate the functioning of prefrontal areas as well as the functional balance between cortical and subcortical functioning could have a good clinical potential (Dalton et al., 2017).

The importance of the network functional architecture in modulating specific cognitive and psychopathological traits of AN is confirmed by the correlations between clustering coefficient and CCI and between global efficiency and STAI scores. These correlations suggest that a higher network segregation is associated with a better global processing in AN, and that a lower global network integration is associated with higher anxiety symptoms. The serious metabolic consequences of AN may in part explain these results. In fact, integration characteristics are more expensive, while segregation properties are less energetically costly and therefore more protective (Bullmore and Sporns, 2012). We can hypothesize that high segregation plays a protective role in AN, since it probably allows a more efficient cognitive processing, but we can also speculate that a lower integration may predispose to higher anxiety symptoms in the disorder. This finding supports the importance of therapeutically addressing anxiety symptoms when they occur in comorbidity with AN (Lloyd et al., 2019; Martín et al., 2019).

The opposite correlation between lowest lifetime BMI and characteristic path length in the experimental group and in the HC one suggests that starvation induced a reduced path length as a form of compensation to minimize the impact of malnutrition, while in healthy subjects, in the absence of malnutrition, a similar effect is observed with an increased BMI.

The relationships between brain structural differences in the two groups and the degree of cortical and subcortical nodes suggest that structural brain alterations in AN have a specific impact on the functional architecture of the brain network. In particular, the observation that these relationships have different directions in cortical and subcortical areas indicates that the functional centrality of these regions may be differently affected by brain atrophy in AN. The increase of degree along with the average decrease in volume was especially observed in nodes of the parietal lobe, including brain areas particularly involved in somatosensory functions and spatial reasoning (Favaro et al., 2012).

Our results also pointed out a role of the 5-HTTLPR polymorphism in modulating the configuration of the overall functional network in AN. Specifically, the low functioning genotype conferred lower small-world properties and a lower modularity in patients with AN, compared with high functioning genotype.

Small-world properties reflect the presence of a proper balance between integration and segregation characteristics of a network, and indicate how much a graph can use local connections to process local information and a small number of long-distance connections to synchronize the overall flow of information (Bassett and Bullmore, 2006). The presence of a peculiar impact of 5-HTTLPR polymorphism on the network architecture suggests a role of serotonin system in balancing segregation and integration properties of the functional connectome in AN. Several lines of evidence show that abnormal activity of the 5-HT system might be involved in AN neurobiology (Solmi et al., 2016). The involvement of the serotonin transporter gene polymorphism in determining the balance of the overall functional connectome in AN could contribute in explaining why the modulation of serotonin pathways is implicated in many processes that are involved in AN etiology, psychopathology, connectivity, and executive functioning (Baker et al., 2018; Teresa et al., 2019). In addition, the opposite role of the polymorphism in AN patients and healthy women might suggest an interaction between this polymorphism and malnutrition in determining the effects on brain network properties.

The present study has two main points of strength. First, it is one of the first studies that investigates the functional connectome in AN by means of graph theory tools and the first to report about hubs distribution. Second, it investigates the role of 5-HTTLPR polymorphism in determining the properties of the network, highlighting the role of serotonin pathways in modulating network architecture. However, some limitations should be mentioned as well. The main limitation is the small sample size, which is due to the pilot nature of the study. Secondly, the study has a cross-sectional design; thus, any casual or prognostic consideration is not allowed.

## Conclusion

In conclusion, our results point out that hub regions are differently distributed in the functional connectomes of patients with AN and HC. The distribution of these regions, alongside the correlation between some of them and specific cognitive functions in AN, suggests that they may play an important role in AN neurobiology. The correlations that emerged between different degree values of nodes and differences in brain volumes suggest an impact of structural alterations on the functional network architecture, also highlighting the need of further longitudinal observations to better understand the role of malnutrition and weight loss on graph metrics. Furthermore, the opposite effects of 5-HTTLPR polymorphism on global network characteristics in the two groups suggest an interaction of the short allele with malnutrition in modulating brain network properties.

## Data Availability Statement

The raw data supporting the conclusions of this manuscript will be made available by the authors, without undue reservation, to any qualified researcher.

## Ethics Statement

Ethical permission was obtained from the Ethics Committee of the Hospital of Padova. After completely describing the study to the subjects, informed written informed consent was obtained.

## Author Contributions

EC and AF: conceptualization, formal analysis, and methodology. EC, AF, and RM: data curation. AF: supervision. EC and PM: writing original draft. AF, ET, and MS: writing, review, and editing.

## Conflict of Interest

The authors declare that the research was conducted in the absence of any commercial or financial relationships that could be construed as a potential conflict of interest.

## Footnotes

1 http://afni.nimh.nih.gov/afni

2 http://www.fmrib.ox.ac.uk
