# Supplementary material for: Functional Connectivity Patterns and the Role of 5-HTTLPR Polymorphism on Network Architecture in Female Patients With Anorexia Nervosa
Source: Front Neurosci. 2019 Oct 14;13:1056. doi: 10.3389/fnins.2019.01056 (PMC6802575; doi:10.3389/fnins.2019.01056)
Supplement: Supplementary file 1 [file Table_1.DOCX]

**Supporting Information**

**Supplementary Methods**

fMRI image analysis

All functional and anatomical images were visually inspected for quality. In order to remove any stabilisation effects, the first five volumes of every scan were discarded. Preprocessing was performed using the Analysis of Functional NeuroImages (version AFNI_2010_10_19_1028; <http://afni.nimh.nih.gov/afni>; NIMH, Bethesda, Maryland) and FM-RIB Software Library (version FSL 4.1.6; http://[www.fmrib.ox.ac.uk](http://www.fmrib.ox.ac.uk); FMRIB, Oxford, UK) tools as described in Biswal et al. (2010). It consisted of motion correction using Fourier interpolation (volume registration using least squares alignment of three translational and three rotational parameters), spatial smoothing with a 6-mm FWHM Gaussian kernel, mean-based intensity normalisation of all volumes by the same factor, linear and quadratic detrending, and spatial normalisation via estimation of a linear transformation (affine with 12 degrees of freedom, using FLIRT, FSL) from the individual functional spaces to MNI152 standard brain space using each individual’s high-resolution anatomic image. A high-pass filter setting of 200 sec (<0.005 Hz) was used to reduce very-low frequency artifacts, such as scanner draft and a low-pass filter to remove any components in the high-frequency spectrum (0.1 Hz). Nuisance signals (6 motion parameters, white matter, cerebrospinal fluid, and the global signal) were removed by multiple regression before performing functional connectivity analyses.

**Supplementary Results**

**Table 1.** Network properties in patients with AN according to 5-HTTLPR genotype, Area Under the Curve (AUC) analysis

|  | Patients with AN  S allele  (n=29) | Patients with AN  LL genotype  (n=7) | Area Under the Curve (AUC) analysis |
| --- | --- | --- | --- |
|  | mean (SD) | mean (SD) | p |
| Assortativity | 0.30 (0.12) | 0.30 (0.10) | 0.935 |
| Global Efficiency | 0.48 (0.05) | 0.49 (0.08) | 0.785 |
| Clustering | 0.61 (0.04) | 0.62 (0.05) | 0.680 |
| Modularity | 0.27 (0.09) | 0.35 (0.06) | 0.160 |
| Path length | 2.18 (0.12) | 2.10 (0.27) | 0.139 |
| Sigma | 1.29 (0.29) | 1.64 (0.32) | 0.028 |

**Table 2.** Network properties in HC according to 5-HTTLPR genotype, Area Under the Curve (AUC) analysis

|  | HC  S allele  (n=29) | HC  LL genotype  (n=7) | Area Under the Curve (AUC) analysis |
| --- | --- | --- | --- |
|  | mean (SD) | mean (SD) | p |
| Assortativity | 0.30 (0.12) | 0.30 (0.10) | 0.261 |
| Global Efficiency | 0.48 (0.05) | 0.49 (0.08) | 0.774 |
| Clustering | 0.61 (0.04) | 0.62 (0.05) | 0.163 |
| Modularity | 0.27 (0.09) | 0.35 (0.06) | 0.071 |
| Path length | 2.18 (0.12) | 2.10 (0.27) | 0.429 |
| Sigma | 1.29 (0.29) | 1.64 (0.32) | 0.051 |

**Fig.1** Correlation between changes in cortical thickness and degree/betweenness values of brain nodes

REFERENCES

Biswal, B. B., Mennes, M., Zuo, X.-N., Gohel, S., Kelly, C., Smith, S. M., et al. (2010). Toward discovery science of human brain function. *Proc. Natl. Acad. Sci.* 107, 4734 LP-4739. doi:10.1073/pnas.0911855107.
